# Enhanced organic photovoltaic-based retinal prosthesis using a cathode-modified structure with plasmonic silver nanoparticles: a computational study

**DOI:** 10.3389/fncel.2024.1385567

**Published:** 2024-05-30

**Authors:** Ali Rahmani, Kyungsik Eom

**Affiliations:** ^1^Department of Electronics Engineering, College of Engineering, Pusan National University, Busan, Republic of Korea; ^2^Department of Electronics, College of Electrical and Computer Engineering, Yadegar-e-Imam Khomeini (RAH) Shahre Rey Branch, Islamic Azad University, Tehran, Iran

**Keywords:** organic photovoltaic cell, photovoltaic interface, retinal prosthesis, silver nanoparticles, cathode nanoparticles, PCPDTBT:PCBM, localized surface plasmon resonance

## Abstract

**Introduction:**

Organic interfaces have recently emerged as a breakthrough trend in biomedical applications, demonstrating exceptional performance in stimulating retinal neuronal cells owing to their high flexibility and compatibility with tissues. However, the primary challenge associated with organic photovoltaics is their low efficiency compared to that of their inorganic counterparts. Among different approaches, embedding plasmonic metal nanoparticles (NPs) in active or buffer layers can efficiently improve photovoltaic cell performance.

**Methods:**

A cathode decorated with silver nanoparticles is introduced to increase the absorption Phenomenon and improve the interface performance as a computational study. In addition to embedding spherical silver nanoparticles in the active layer (A-AgNPs), a monolayer array of spherical AgNPs in the cathode electrode (K-AgNPs) is incorporated. In this configuration, the large K-AgNPs play dual roles: acting as cathode electrode and serving as plasmonic centers to increase light trapping and absorption. The bulk heterojunction PCPDTBT:PCBM is chosen as the active layer due to its favorable electronic properties.

**Results:**

Our computational analysis demonstrates a notable 10% enhancement in the photovoltaic cell current density for the developed structure with K-AgNPs in contrast to without them. Additionally, the simulation results reveal that the modeled device achieves a two-fold efficiency of the bare photovoltaic cell (without A-AgNPs and K-AgNPs), which is particularly evident at a low intensity of 0.26 mW/mm^2^.

**Discussion:**

This study aims to propose an efficient epiretinal prosthesis structure using a different strategy for plasmonic effects rather than conventional methods, such as incorporating NPs into the active or buffer layer. This structure can prevent the harmful side effects of using large metal NPs (*r* > 10 nm) in the active layer during exciton quenching, charge trapping, and recombination, which deteriorate the power conversion efficiency (PCE).

## 1 Introduction

Retinal degenerative diseases, such as retinitis pigmentosa (RP) and age-related macular degeneration (AMD), can result in vision loss owing to the progressive deterioration of photoreceptors. RP is the primary cause of inherited blindness in young individuals, and it currently lacks effective treatment. AMD is the predominant cause of irreversible blindness in the elderly (Mandel et al., 2013). In this vision loss scenario, the inner retinal neurons retain the ability to process visual signals and transmit them to the brain. Therefore, patterned electrical stimulation of these neurons holds promise for creating pattern perception and for partial vision restoration in visually impaired individuals (Zrenner et al., 2011; Humayun et al., 2012).

Various strategies are employed to convey visual signals to retinal neurons, with the primary approach being electrical stimulation achieved through electrode arrays positioned in either the epiretinal or the subretinal space (Lorach et al., 2015). Although both techniques have been approved for clinical use (Lorach et al., 2015), they require intricate surgical procedures that involves the implantation of electrodes with trans-scleral cables. Notably, the visual acuity of the epiretinal system (ARGUS II, Second Sight Inc., United States) is approximately 20/1260 and perceptual distortions are observed due to the axonal stimulation (Nanduri et al., 2012). More importantly, the use of inductive link antennas, ASIC chips, and other circuits for pulse generation constitute bulky devices, thereby severely damaging the eyes during implantation. Additionally, the restricted number of channels contributes to low visual acuity and prevents the achievement of normal vision.

Over the past decade, a novel approach has emerged in which photovoltaic (PV) pixels convert incident light into an electric current without requiring bulky implants for pulse generation (Mathieson et al., 2012; Wang et al., 2012). Unlike the previous systems, wherein implanted retinal stimulation electrodes were linked via trans-scleral cables to an electronic data box, which in turn connected to an external camera through a receiving coil and inductive telemetry (Mandel et al., 2013), in this system, the input light pulses are projected from video goggles into a PV array of silicon photodiodes and converted into electrical pulses to stimulate retinal neurons. This interface addresses the numerous drawbacks associated with ARGUS II while incorporating several advantageous features. Notably, its compact size compared with other techniques eliminates the need for an inductive system, data transfer channels, and a high level of vision quality. Many pixels can be wirelessly activated by incident light, making it less invasive than the ARGUS II device. However, susceptibility to fracture under mechanical pressure as well as tissue damage including inflammatory reaction due to mechanical mismatch are other issues faced by silicon-based photodiodes which effectively addressed the employing organic materials (Green and Abidian, 2015; Antognazza et al., 2016).

Organic materials have been proven to be the preferred choice for biomedical interfaces and neuronal activation due to having several advantages to convert incident light into electrical stimulation signals (Martino et al., 2015; Feyen et al., 2016). These key advantages of organic structures include their biocompatibility, low cost, lightweight, and flexibility, which position them favorably over silicon structures in biomedical devices. Their inherent flexibility and bendability make them suitable for conforming to the exact curvature of the retinal space (Ghezzi et al., 2011). Currently, organic semiconductors such as poly(3-hexylthiophene-2,5-diyl), P3HT, and ([6,6]-phenyl-C61-butyric acid methyl ester), PC_61_BM, utilized as bulk heterojunction (BHJ) in P3HT:PC_61_BM, as well as a conjugated polymer like [poly(3,4-ethylenedioxythiophene)-polystyrene sulfonate], PEDOT:PSS, are focal points in the development of retinal prostheses by various research groups (Maya-Vetencourt et al., 2017). In this regard, Ferlauto et al. (2018) used P3HT:PCBM as an active layer for retinal prosthesis and light wavelength of 565 nm with intensity of 943.98 μW/mm^2^ which led to 200 μA/cm^2^ current density. Also, Chenais et al. (2021) irradiated the same active layer with wavelength of 565 nm and intensity of 0.9 mW/mm^2^ which caused a current density of 10 μA/mm^2^. However, the low efficiency of these structures is major challenge, limiting device miniaturization and thereby the worsening the visual acuity (Rathbun et al., 2014). Miniaturizing the photovoltaic cell size without efficiency enhancement or high incident light intensity leads to a decrease in the number of generated free carriers, subsequently resulting in lower stimulating current within the photovoltaic cell, which may fail to activate the neurons.

Scholars have explored two distinct approaches to achieve a more efficient structure: (1) Modifying the materials of the active layer. They have turned to the use of poly [2,6-(4,4-bis-(2-ethylhexyl)-4H-cyclopenta [2,1-b;3,4-b′]dithio-phene)-alt-4,7(2,1,3-benzothiadiazole)] blended with PCBM, known as PCPDTBT:PCBM bulk heterojunction due to its ideal bandgap, excellent absorption, and superior charge carrier mobility compared to P3HT:PCBM resulting in enhanced performance in photovoltaic cells (Rahmani and Eom, 2023). (2) Employing quantum dots and metal nanoparticles. Leccardi et al. presented a retinal interface based on PCPDTBT:PCBM active layer and irradiation of 565 nm light wavelength with intensity of 1 mW/mm^2^ which gained a current density of 10 μA/mm^2^ (Airaghi Leccardi et al., 2020). Other research groups employed quantum dots to improve the function of organic bio-interfaces. Karatum et al. (2022) presented a flexible colloidal quantum dot-based photovoltaic interface using NIR light. They applied a 10 ms pulses of 780 nm wavelength with a 1 mW/mm^2^ incident light power which provides 550 μA/cm^2^ as maximum current density. In the other work they used P3HT:ITIC active layer to provide neurodegenerative and neuroprotective effects (Kaleli et al., 2024). In this case, they incorporated ZnO nanoparticles as electron transport layer which benefits NIR light and low cost. Over the last decade, plasmonic nanoparticles have emerged as a crucial approach in the field of PV with the aim of improving photovoltaic cell efficiency (Tan et al., 2012; Wang et al., 2018). Metal nanoparticles can affect organic photovoltaic (OPV) performance by increasing optical absorption based on the optical path length owing to the enhanced scattering and electromagnetic field strength through near-field enhancement arising from localized surface plasmon resonance (LSPR) excitation (Ahn et al., 2016). However, low efficiency remains a crucial challenge for these modified materials and needs to be addressed. Additionally, embedding metal nanoparticles in a large volume, despite increasing carrier generation, can damage the active layer function.

In this paper, we propose an efficient organic photovoltaic cell structure for epiretinal prostheses. To enhance the efficiency, we propose the use of a monolayer array of spherical silver cathode nanoparticles. These cathode nanoparticles (K-AgNPs) serve as both cathode and plasmonic resonant nanoparticles. Employing this technique increases the total absorption and results in a higher efficiency compared to a photovoltaic cell without K-AgNPs. The rationale behind opting for silver lies in its dual applicability, possessing both bio-metallic properties suitable for plasmonic nanoparticles and an appropriate work function as a cathode. We use PCPDTBT:PCBM as the active layer, in which spherical silver nanoparticles (A-AgNPs) are incorporated. We examined the impact of key structural factors, such as the radius and filling fraction (f_s_) of the A-AgNPs on the performance of the interface. The size and interparticle gap of the K-AgNPs are tuned to have the same plasmonic resonance wavelength as the A-AgNPs in the active layer, aligned to the incident light wavelength (453 nm). Finally, considering the threshold conditions for activating neurons, the designed interface successfully stimulated retinal neurons. This efficient structure can be powered by a lower light intensity, making it a promising neural interface for future retinal prosthetics.

## 2 Materials and methods

### 2.1 Photovoltaic cell structure modeling using cathode-modified structure

#### 2.1.1 Photovoltaic structure modeling

**Figure 1:**
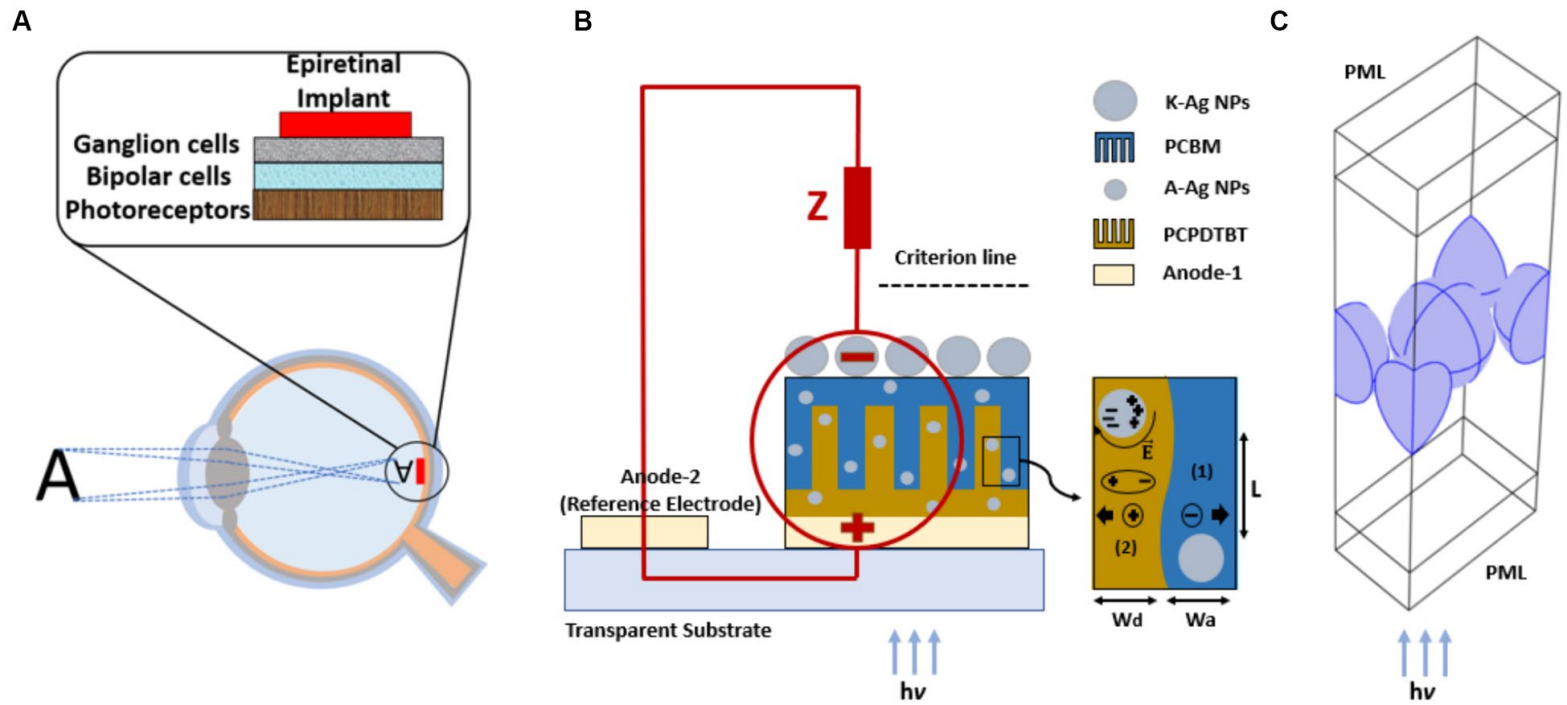
Implanted epiretinal interface in the retina space **(A)**, AgNPs as cathode electrode (K-AgNPs) perform as both cathode electrode and plasmonic sources and stimulate unmyelinated neuron (circuit model), the embedded A-AgNPs in the comb shape active layer, and the simulated photovoltaic unit cell with exciton generating process **(B)**, rectangular unit cell with periodic boundary conditions and perfectly matched layer (PML) in the electromagnetic modeling of K-AgNPs **(C)**.

To enhance the efficiency of the photovoltaic cells, we used PCPDTBT and PCBM as the active layers because they are known to be efficient low-bandgap photovoltaic materials (Peet et al., 2007; Soci et al., 2007; Morana et al., 2008). To model the epiretinal photovoltaic cell interface (Figure 1A), a two-dimensional (2D) periodic comb shape is modeled to reflect the BHJ active layer. As the modeled active layer is periodic and comprised intertwined segments of donor-acceptor domains, we simulated one fragment with curvature formation at the donor-acceptor interface (Figure 1B) (Raba et al., 2013). In the model, L, W_d_, and W_a_ represent the active layer thickness and average widths of the donor and acceptor layers, respectively. Regarding the basic crystal cell dimensions of PCPDTBT and PCBM in the BHJ blend and the donor and acceptor morphologies, the mean values of W_d_ and W_a_ are 14 and 25 nm, respectively. These values coincide with the PCPDTBT:PCBM volume ratio of 1:1.8, considering the density and weight ratio of 1:3 (Prosa et al., 1992; Stratakis and Kymakis, 2013). Additionally, the suitable thickness of the active layer, L, is 70 nm (Boland et al., 2010).

One fragment of the active-layer structure is modeled and simulated using COMSOL Multiphysics (COMSOL Inc., Burlington, MA, United States). The simulation employed a two-dimensional domain, semiconductor module, and extra-coarse meshing. The complex refractive indices of the materials are incorporated as functions of the input light wavelength (Klein et al., 2015). The donor and acceptor materials are introduced into COMSOL, and their properties are inserted based on experimental work (Schulz et al., 2017).

#### 2.1.2 Plasmonic A-AgNPs modeling

Metal NPs have been extensively investigated as plasmonic structures due to their LSPR which can be controlled by manipulating their shape and size (Ringe et al., 2010). When these metal NPs are embedded in photovoltaic cells, both the near-field effect of the surface plasmons and the enhanced scattering contribute to an overall improvement in absorption within the UV–Vis region. Near-field surface plasmons exhibit high efficacy in concentrating the electric field at the interface between metal NPs and dielectric medium. The scattering section increased the effective optical path length beyond the physical thickness of the absorbing layer.

For NPs with dimensions below the wavelength of the incident light, the absorption (C_abs_) and scattering (C_scat_) cross-section areas in the quasi-static limit are described as Bohren (1983),


(1) $$C_{\mathrm{abs}}=\frac{2\pi }{\lambda }Im\left[\alpha_{sp}\right]$$

(2) $$C_{\mathrm{scat}}=\frac{1}{6\pi }{\left(\frac{2\pi }{\lambda }\right)}^{4}{\left|\alpha_{sp}\right|}^{2}$$

(3) $$\alpha_{sp}=3V\frac{\varepsilon_{m}-\varepsilon_{s}}{\varepsilon_{m}+2\varepsilon_{s}}$$

where, C_abs_ is proportional to the imaginary part of $\alpha_{sp}$ , in which $\alpha_{sp}$ denotes the NP polarizability, V indicates the volume of NP, which is 4/3πr^3^ and r indicates the radius of the NP, λ represents the wavelength of incident light, and $\varepsilon_{m}$ and $\varepsilon_{s}$ represent the wavelength-dependent dielectric functions for the metal NPs and surrounding medium, respectively. In terms of the equations that govern the absorption and scattering cross sections, the scattering term is significantly more dependent on the particle size than the absorption term. The normalized absorption (Q_abs_) and scattering cross section (Q_scat_) are considered by the ratio of their terms and cross-section area of NPs, which is 𝜋r^2^, i.e., Q_abs_ is C_abs_/𝜋r^2^ and Q_scat_ is C_scat_/𝜋r^2^. The scattering efficiency (Q_sc_) is given as Bohren (1983):


(4) $$Q_{sc}=\frac{C_{\mathrm{scat}}}{C_{\mathrm{scat}}+C_{\mathrm{abs}}}$$

Q_sc_ represents the ratio of scattering to the absorption cross-sectional area. The scattering enhancement is more pronounced for large NPs, whereas it becomes negligible for small NPs. In contrast, near-field enhancement is prevalent for smaller-diameter NPs, particularly those <50 nm in size (Stratakis and Kymakis, 2013). For the wavelength at which the relative permittivity of metal and medium is such that $\varepsilon_{m}=-2\varepsilon_{s}$ , the polarizability of NPs and consequently the absorption cross-section becomes maximal, and the localized surface plasmon resonance condition occurs. LSPR is dependent on the shape, size, material, and surrounding medium of the NPs. Selecting a larger size and f_s_ of A-AgNPs greater than 10 nm in radius and 10% in f_s_ damages the active layer function, causing exciton extinction and free charges trapping which decrease the efficiency of the device (You et al., 2014).

#### 2.1.3 Far-field electromagnetic modeling of AgNPs array in cathode electrode (K-AgNPs)

Plasmonic metal NPs can be employed as effective light-trapping sources, positioned either between the layer interfaces or within the buffer or active layer of a photovoltaic cell, to enhance photon absorption (Otieno et al., 2017). We introduce a monolayer array of spherical AgNPs instead of a conventional cathode plate which serves a dual purpose: first, it facilitates the injection of the current required to activate the neurons as a cathode; second, it acts as a plasmonic source, increasing the total light absorption. The radius of these cathode nanoparticles (K-AgNPs) is intentionally larger than that of A-AgNPs embedded in the active layer, resulting in higher absorption as the plasmonic source. This approach avoids the several disadvantages associated with using large metal nanoparticles (*r* > 10 nm) in the active and buffer layers, such as exciton extinction, charge trapping, and recombination, which can impede the efficient transfer of charge carriers to the cathode. Large metal nanoparticles block and trap free carriers during carrier movement through the active layer toward the cathode and quench the excitons by electron transfer (You et al., 2014).

Computational far-field electromagnetic modeling for the solution of Maxwell’s equation is conducted using the finite element method (FEM) solver within the electromagnetic wave module of COMSOL Multiphysics in the frequency domain (ewfd). In the simulation, an infinite array of K-AgNPs is exposed to incident electromagnetic waves in a normal direct-incidence configuration. Electromagnetic modeling assumes infinite 2D arrays using a unit cell and considers periodic boundary conditions on the side walls. An NP-centered rectangle with four quarter slices of spherical NP, each oriented at an angle of 30° to the central NP, is constructed as a unit cell model (Figure 1C). The light waves are incident from the bottom (active layer) and exit from the top of the device (K-AgNPs), interfacing with the retinal tissue. The surrounding medium for the K-AgNPs is water, which has a refractive index of 1.33. A perfectly matched layer (PML) is assumed at the top and bottom of the boundaries to ensure total absorption of light waves (Borah et al., 2022). The refractive index (n) and extinction index (k) of the AgNPs as a function of the incident wavelength are obtained from Palik (1998). Meshing accuracy is confirmed by using varying meshes and element sizes, with an extremely fine meshing type ultimately selected to optimize the simulation precision.

In this case, the radius of the K-AgNPs is 25 nm (R), and the interparticle gaps (d) are assumed to be 1, 1.5, 2.5, and 5 nm to determine the resonance wavelength, matching that of the A-AgNPs embedded in the active layer. The selection of a 25 nm radius can be attributed to having a larger NP compared to the A-AgNP to gain higher absorption, owing to the restriction that the K-AgNP size should be much smaller than the incident light wavelength. Notably, quantum effects are negligible for nanoparticles with a radius of 25 nm and an interparticle gap of 1 nm or larger (Kreibig, 1995). Barrow et al. demonstrated that even for an interparticle gap as small as 0.5 nm, there exists acceptable compatibility between classical electromagnetic assumptions and experimental results. This is attributed to the negligible impact of the quantum tunneling effects under such conditions (Barrow et al., 2012).

### 2.2 Light intensity attenuation in photovoltaic cell structure using beer–Lambert’s law

As light travels along the active layer before reaching the K-AgNPs, its intensity decays owing to the scattering and absorption within the active layer. Subsequently, the light intensity that reaches the K-AgNPs is calculated using the Beer–Lambert law, which incorporates the attenuation due to the A-AgNPs and PCPDTBT:PCBM layers. The Beer–Lambert law establishes a relationship between light attenuation and the material properties encountered by the light. The standard equation for absorption is expressed as follows (Protasenko et al., 2006):


(5) $$A=\log\frac{I_{0}}{I}$$

where *A* indicates the amount of light absorbed by the sample at a given wavelength, and *I*_0_ and *I* (W/m^2^) denote the input and output light intensities, respectively. For A-AgNPs with an absorption resonance wavelength, molar absorptivity is used to quantify how a material absorbs light and determines light transmission through the A-AgNPs over the incident wavelengths. In this case, the absorption is given by


(6) $$A=\alpha l=\left(\epsilon c\right)l$$

where *α* is the absorption coefficient, *ϵ* (LM^−1^ cm^−1^) represents the molar absorptivity, *l* (cm) indicates the distance the light travelled in the material, and *c* (ML^−1^) is the concentration of the absorbing material per unit volume. Molar absorptivity (ϵ) can be calculated from the absorption cross-section σ (cm^2^) spectrum as follows (Protasenko et al., 2006):


(7) $$\in =\frac{N_{A}}{2.3\times 10^{3}}\sigma$$

where *N_A_* is Avogadro’s number, and *σ* is C_abs_/N, in which, C_abs_ (m^2^) is obtained from Eq. 1 and *N* is the number of atoms per A-AgNP. Using Eq. 5 and 6, the total absorption and transmission for A-AgNPs is calculated as $1-10^{-\in \mathrm{cl}}$ and, $10^{-\in \mathrm{cl}}$ , respectively. The light transmission through the bare active layer is calculated by $e^{-\alpha x}$ , in which x denotes the length of the active layer, *α* is the absorption coefficient, which equals $\frac{4\pi }{\lambda }k\left(\lambda \right)$ , where *k* indicates the imaginary part of the refractive index (extinction coefficient) of PCPDTBT:PCBM versus wavelength (Klein et al., 2015).

### 2.3 Current generation in the embedded active layer

Upon photoexcitation of the organic material, singlet excitons are formed, which subsequently diffuse to the donor-acceptor interface within their respective lifetimes (Ghezzi et al., 2011). When metal NPs are embedded in a photovoltaic cell, the generation of charge carriers is enhanced primarily by the near-field effects that result from the LSPR phenomenon. Although its contribution is minimal compared to the near-field effects, far-field scattering also contributes to an increased carrier generation rate. Free electrons arising from this process generate a higher current compared to the bare active layer, i.e., the active layer without A-AgNPs (Wang et al., 2011). For the bare active layer, the short-circuit current density (J_sc_) is calculated using the generation rate (G) of the free charges (Eqs. 8, 9) (Wu et al., 2011; Piralaee et al., 2020).


(8) $$G\left(\lambda \right)=\alpha \left(\lambda \right)\lambda I_{0}P/hc$$

(9) $$J_{sc}\left(\lambda \right)=G\left(\lambda \right)qL$$

where *α* is the absorption coefficient and equals $\frac{4\pi }{\lambda }k\left(\lambda \right)$ , *k* is the extinction coefficient of the medium, λ is the wavelength of the incident light, *h* is Planck’s constant, *c* is the speed of light, P denotes the probability of excitons dissociation of the active layer material at the interface, L is the active layer height, and *q* is the electron charge. Here, *I*_0_ represents the light intensity determined using the Beer–Lambert equation, as described in Section 2.2.

When applying metal NPs to the active layer, the enhancement in J_sc_ due to the absorption and scattering effects of the metal NPs should be considered. Hence, it is essential to consider the J_sc_ generated by the bare active layer as well as the impact of the active layer adjacent to the A-AgNPs and the active layer in the vicinity of the K-AgNPs. The experimentally determined *p* value for the bare active layer is 0.7 and for the active layer around the A-AgNPs and K-AgNPs is 0.85 (Yamamoto et al., 2012; Lu et al., 2013). In calculating J_sc_, the scattering term for both the A-AgNPs and K-AgNPs is ignored due to their small size (≤50 nm), and only the absorption term is considered. Furthermore, because the influence of metal NPs on the active layer is limited to a constrained region around the NP, the absorption cross-section area, we employed a volume ratio that represents the extent of the impact of the field enhancement on the active layer. Regarding the bare active layer function in Section 2.1.1, the effect of plasmonic A-AgNPs discussed in Section 2.1.2, and K-AgNP analysis in Section 2.1.3, J_sc_ is represented as in Eq. 10:


(10) $$J_{sc}\left(\lambda \right)=\left(I_{0}\lambda qL/hc\right)\left[\begin{array}{l} \left(70\%\alpha_{1}\left(\lambda \right)\left(1-f_{s}-v_{1}f_{s}-v_{2}\right)\right)+\\ \left(85\%\alpha_{2}\left(\lambda \right)\left(1-Q_{sc}\right)\left(v_{1}f_{s}\right)\right)+\\ k\left(85\%\alpha_{2}\left(\lambda \right)\left(v_{2}\right)\right) \end{array}\right]$$

where *α*_1_ and *α*_2_ are the absorption coefficients of PCPDTBT:PCBM and A-AgNPs as a function of incident light wavelength, respectively. 1-Q_sc_ is the near-field absorption efficiency of A-AgNPs, f_s_ corresponds to the filling fraction of A-AgNPs, and v_1_f_s_ and *v*_2_ indicate the absorption term volumes of the A-AgNPs and K-AgNPs, respectively, in contrast to the volume of the active layer. In this case, *v*_1_ is the ratio of the volume of the near-field absorption cross-section region around the NP (V_abs_) to that of the NP, and v_2_ is the volume ratio of region of the active layer affected by the light absorption of K-AgNPs to the region of active layer. The parameter is computed through the individual K-AgNP absorption cross section obtained by the analysis in Section 2.1.3.

The first term of J_sc_ is related to the free charges generated in the bare part of the active layer, while the second term represents the carriers generated by the light absorption of A-AgNPs with an absorption probability of (1-Q_sc_). The third term represents the carriers generated owing to the absorption term resulting from K-AgNPs, which occurs in the active layer in the vicinity of the K-AgNPs and includes v_2_. The coefficient of k, which is equal to T_r_ × A_b_, describes that portion of incident light which is absorbed by the K-AgNPs. T_r_ denotes the total transmittance of the incident light through the A-AgNPs and active layer, reaching the K-AgNPs based on Beer–Lambert’s law, and A_b_ indicates the light absorptance of K-AgNPs, which is computed from the far-field optical analysis of the K-AgNP array using electromagnetic modeling. Some commonly used acronyms and abbreviations in the text with their definitions and the modeling parameters of the structure are listed in Tables 1, 2, respectively.

**Table 1:** List of abbreviations.

No.	Acronym/Abbreviation	Definition
1	NPs	Nanoparticles
2	A-AgNPs	Spherical silver nanoparticles in the active layer
3	K-AgNPs	Spherical silver nanopaticles as the cathode electrode
4	PCE	Power conversion efficiency
5	f_s_	Filling fraction (for A-AgNPs)
6	LSPR	Localized surface plasmon resonance
7	ewfd	Electromagnetic wave in the frequency domain
8	J_sc_	Short-circuit current density

**Table 2:** Modeling parameters for the photovoltaic cell in COMSOL.

Parameter	Definition	Value	Unit
r	A-AgNP radius	5, 7.5 and 10	nm
R	K-AgNP radius	25	nm
d	Interparticle gap of K-AgNPs	1, 1.5, 2.5, 5	nm
f_s_	Filling fraction factor (f_s_)	5, 7.5 and 10%	–
I	Incident light intensity	0.26 and 0.33	mW/mm^2^
A	Photovoltaic cell area	100×100	μm^2^
L	Active layer thickness	70	nm
W_d_	Donor thickness	14	nm
W_a_	Acceptor thickness	25	nm

### 2.4 Neural stimulation by K-AgNPs in plasmonic photovoltaic cells

**Figure 2:**
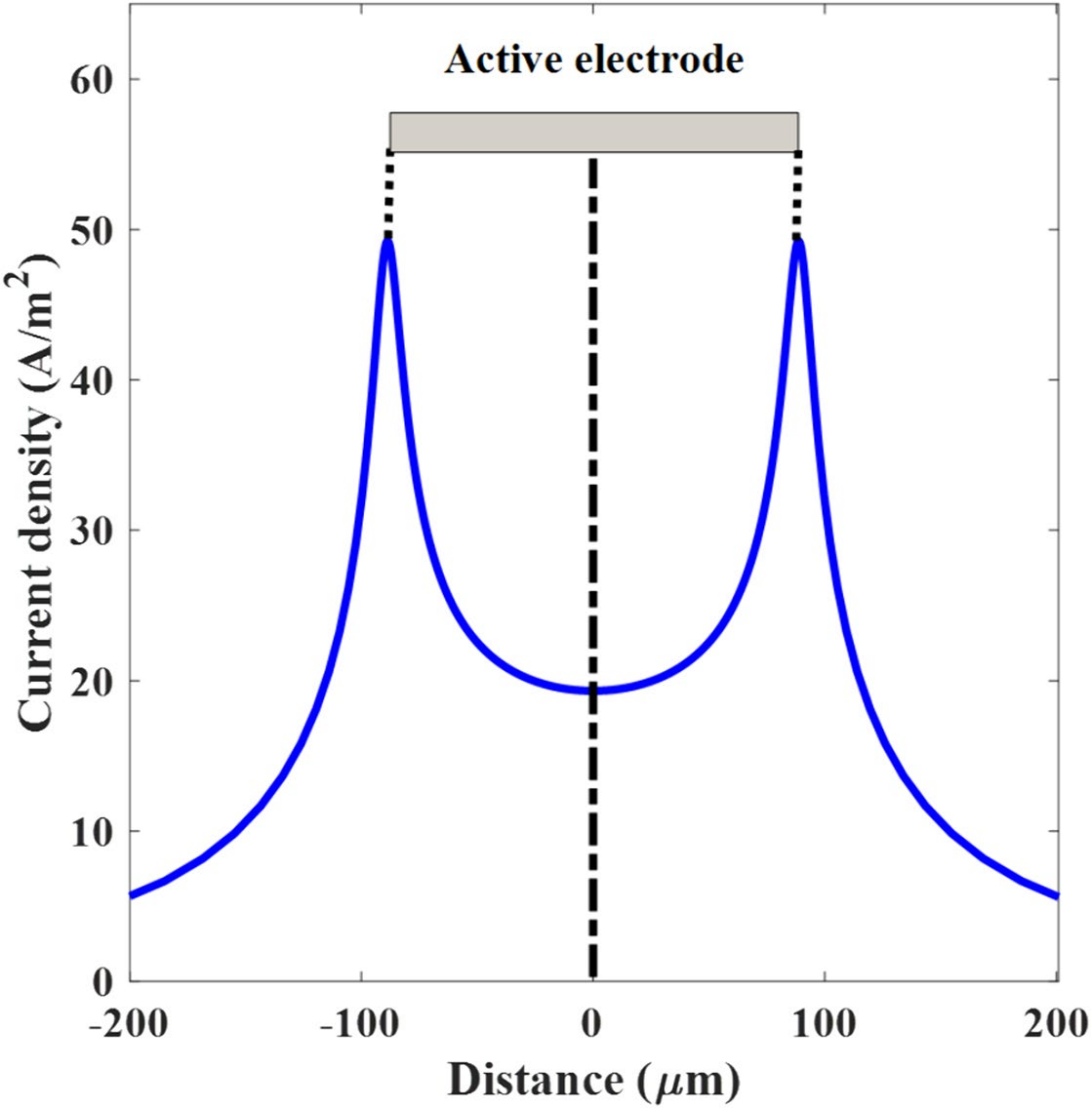
Threshold current density over the distance along the electrode surface. The active electrode on the top of the graph is in the shape of a gray-colored rectangle and centered at 0 μm.

To validate the designed photovoltaic cell featuring AgNPs embedded in both the active layer and the cathode electrode, we modeled an unmyelinated retinal neuron and a stimulating electrode capable of delivering electrical activation pulses using the neuronal dynamics simulator SIM4LIFE LIGHT (Zurich Medtech AG (ZMT), Switzerland) (Rahmani and Eom, 2023) (Figure 2). A voltage source with electrical characteristics identical to those of the designed photovoltaic cell is described in Section 2.3 is applied to the electrode to stimulate neurons.

A spatially extended stimulation model is applied (Rahmani and Eom, 2023). The diameter of the axon (d_x_) is selected within the range of the human retinal axon diameter (FitzGibbon and Taylor, 2012; FitzGibbon and Nestorovski, 2013). The retinal ganglion cells (RGC) are located at the central region of the active electrode, parallel to the electrode, separated by a distance (l) from the surface of the electrode.

In this simulation, monophasic cathodic stimulation is employed and the voltage magnitude is varied to determine the threshold voltage. The model incorporates the active and reference electrode areas (A) with a thickness (t) equal to the diameter of the K-AgNPs, ensuring the representation of electrical activation. For each photovoltaic cell and electrode, the geometric surface area (A) is 100 × 100 μm^2^. However, when considering the overall surface area of the total number of K-AgNPs per photovoltaic cell, the effective surface area of the electrode (A_eff_) expands to 177 × 177 μm^2^.

In this case, the K-AgNPs interface with the neurons, leading to the injection of current into the neurons based on the applied voltage. In this model, the electrodes are assumed to establish perfect contact. The threshold current density is defined as the minimum current required to elicit an action potential. The current density is measured along the active electrode, centered at 0 μm. The measurement of current density is performed along the axonal trajectory, following a specified criterion line, and running parallel to the electrode, at a distance of 5 μm above the active electrode surface (Figure 1B). The spatial distributions of the applied potential and current density are simulated at a frequency of 10 Hz (computational frequency), corresponding to a 100 ms repetition period of applied current density. The computed current density is introduced into the neuron with a cathodic duration pulse of 1 ms, corresponding to a 1 kHz sinusoidal stimulus (Mathieson et al., 2012). The detailed modeling parameters for both the neuron and the photovoltaic cell, along with the corresponding values, are listed in Table 3.

**Table 3:** Modeling parameters in SIM4LIFE LIGHT.

Parameter	Definition	Value	Unit
A	Geometrical surface area of electrode	100 × 100	μm^2^
A_eff_	Effective surface area of electrode	177 × 177	μm^2^
t	Electrode thickness (K-AgNP diameter)	50	nm
l	Electrode surface distance from the neuron	5	μm
d_x_	Axon diameter	1	μm

In computing the current density introduced by the cathode, the average current density across the entire electrode, from edge to edge (−88.5 to +88.5 μm), is 25.1 A/m^2^ (Figure 2). As the electrode is on the top of the retinal ganglion cell (RGC), the threshold current/voltage is determined to stimulate the RGC. However, to stimulate a deeper area of the retina and inner nuclear layer, a higher current is required and consequently not only the deeper layer of retina tissue but also the RGC will be activated. Since the current density has inverse relation with the area, there are two sharp peaks of the current density in the thin edges of the electrodes. The overall impedance of the electrode-neuron system derived from the voltage-to-current ratio (V/I), corresponding to the threshold voltage and current density (−0.11 V and 25.1 A/m^2^, respectively), is 4.3 kΩmm^2^. According to the SIM4LIFE LIGHT material library, the electrical conductivity and relative permittivity values of the tissue at 10 Hz are 0.027 S/m and 4.06 × 10^7^, respectively.

## 3 Results

### 3.1 Plasmonic effect of A-AgNPs in the active layer

**Figure 3:**
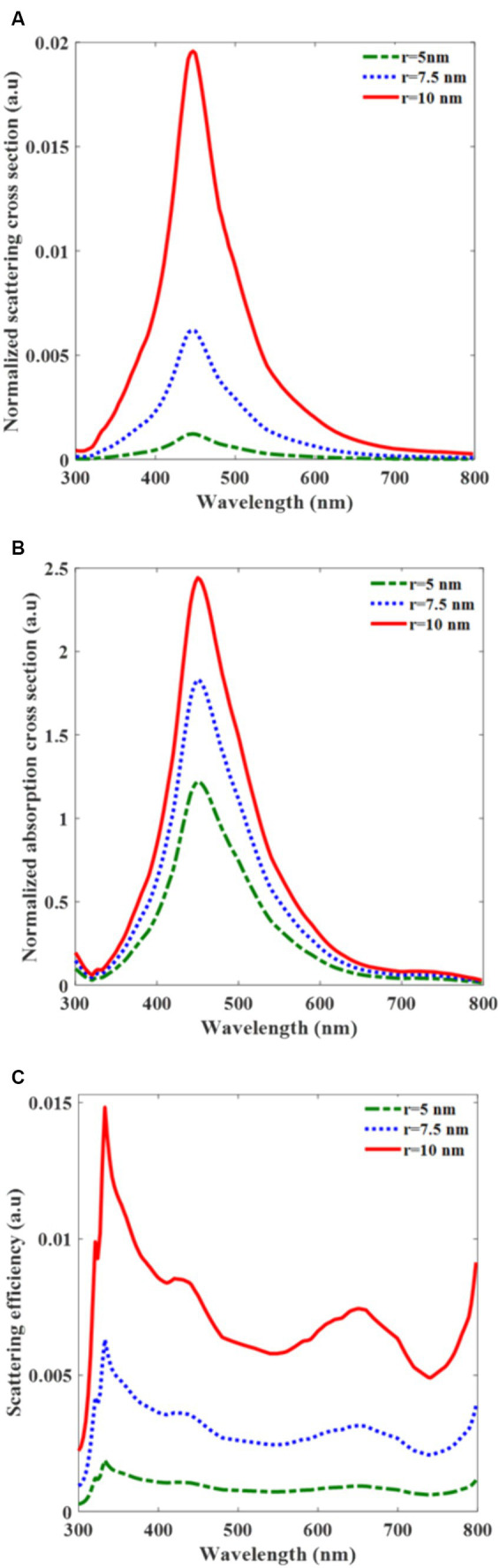
Normalized scattering cross-section for silver nanoparticle (A-AgNP) in the PCPDTBT:PCBM active layer **(A)**, Normalized absorption cross-section **(B)**, and scattering efficiency **(C)** versus the wavelength for different radii of incorporated A-AgNP.

We simulated the scattering and absorption term of A-AgNPs of various sizes over the different wavelengths (Figure 3). Based on the Eqs. 1, 2, both terms are proportional to the polarizability. Regarding to the Eq. 3, at a specific wavelength, the polarizability resonates in which the resonance wavelength depends on the active layer and nanoparticle materials. In Figures 3A,B, the normalized scattering and absorption cross sections show a resonant peak at a wavelength of 453 nm, aligned with the resonance wavelength of the A-AgNPs. Since the polarizability is proportional to the nanoparticle volume (Eq. 3), by increasing the radius of nanoparticle, both normalized scattering and absorption terms are increased. The maximum normalized scattering cross-sectional values for the radii of 5, 7.5, and 10 nm are 0.0015, 0.006, and 0.019 at the wavelength of 453 nm, respectively (Figure 3A). Also, the corresponding normalized absorption cross sections values are 1.2, 1.8, and 2.4, respectively (Figure 3B). For all simulated A-AgNP sizes, the absorption values exceeded the scattering values. Considering Eq. 4, the scattering efficiency is increased by increasing the radius of A-AgNP. In Figure 3C the scattering efficiencies are 0.001, 0.003, and 0.007 for A-AgNP radii of 5, 7.5, and 10 nm, respectively at resonance. From the scattering efficiency, we also confirmed that the maximum value is 0.015 for A-AgNPs with a radius of 10 nm over all wavelengths (Figure 3C). This result further validates that the total light absorption is primarily attributable to the absorption term, rendering the contribution of scattering negligible. The absorption term is necessary to determine parameter v_1_ in J_sc_ in Eq. 10.

### 3.2 Far-field optical properties of cathode nanoparticles

**Figure 4:**
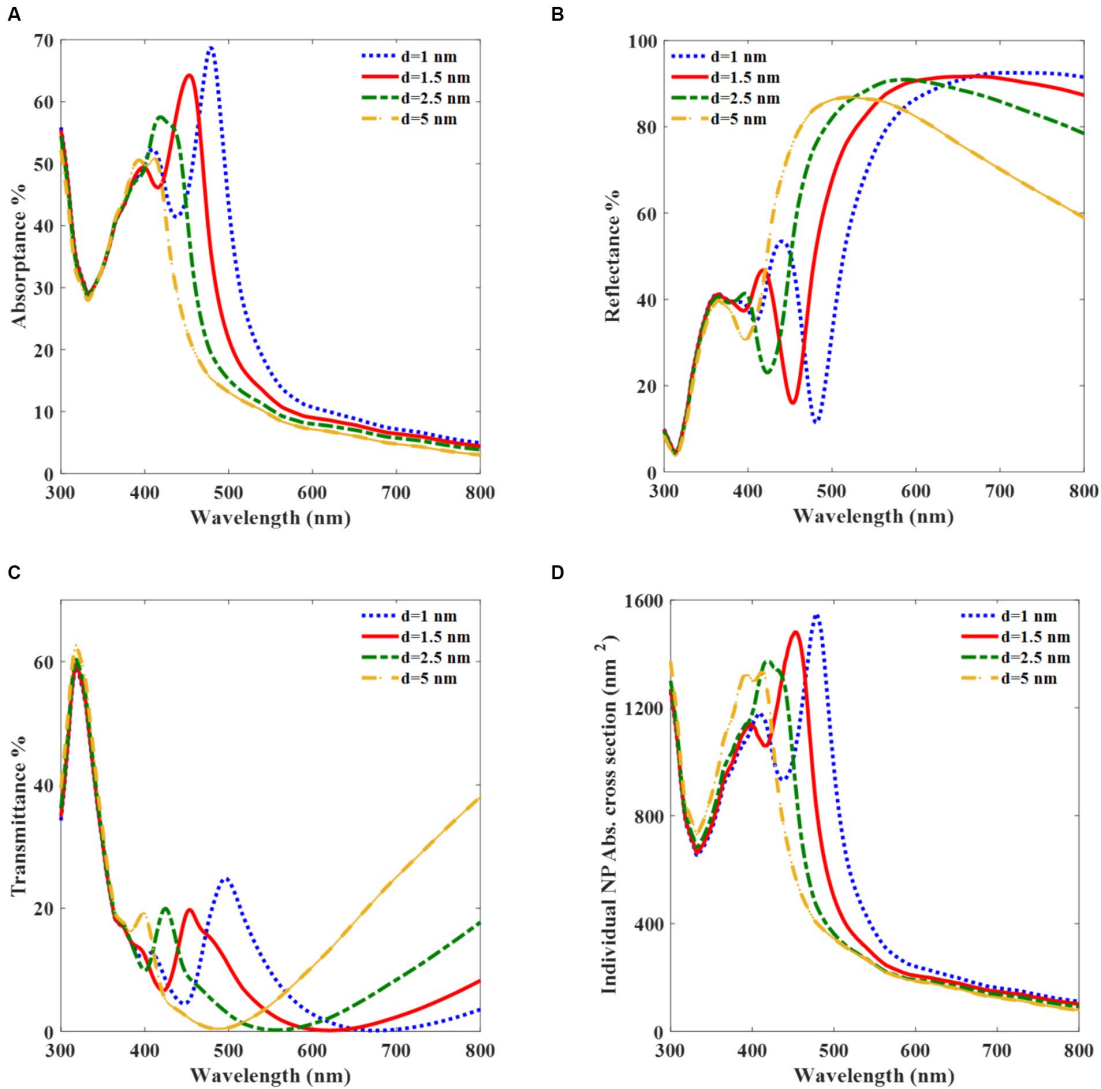
Optical properties of monolayer array of K-AgNPs with a radius of 25 nm in water medium (*n* = 1.33). Absorptance **(A)**, Reflectance **(B)**, Transmittance **(C)**, and absorption cross-section per K-AgNP **(D)**, versus the normal incident light wavelength for different interparticle gaps.

Subsequently, we explore the effects of K-AgNPs, particularly the role of the radius and interparticle gap on their optical properties to enhance charge generation. First, the absorptance properties of K-AgNPs for different interparticle gaps are determined to find the absorbed light by K-AgNPs and hence the generation rate in Eq. 8. The absorptance peaks corresponding to interparticle gaps of 1, 1.5, 2.5, and 5 nm are 68, 65, 58, and 50%, respectively (Figure 4A). This suggests that the absorption spectrum undergoes a blue shift with an increase in the interparticle gap. To enhance the overall light absorption at both K-AgNPs and A-AgNPs, the interparticle gap of 1.5 nm and radius of 25 nm are selected for K-AgNPs to match their resonance wavelength to that of A-AgNPs in the active layer (453 nm). The corresponding absorptance value for this interparticle gap equals to A_b_ in Eq. 10 (k = T_r_ × A_b_). The corresponding values of the local minimum reflectance are identified as 10, 15, 22, and 32% for the interparticle gaps of 1, 1.5, 2.5, and 5 nm, respectively. As the inter-particle gap increases, the reflectance trajectories exhibit a blue shift (Figure 4B). As shown in Figure 4C, the transmittance curve undergoes a blue shift when the K-AgNPs are spaced apart, with peak values of 22, 20, 20, and 18% for interparticle gaps of 1, 1.5, 2.5, and 5 nm, respectively. As a complementary result, Figures 4B,C show that the amplitude of reflectance and transmittance in the resonance wavelength are small and the main part of incident light is absorbed by K-AgNPs. The absorption cross section of the individual particles undergoes a blue shift as the interparticle gap increases. The absorption cross-section become 1,500 nm^2^ when the gap is 1.5 nm at a wavelength of 453 nm (Figure 4D). The value of 1,500 nm^2^ is essential for finding v_2_ when calculating Eq. 10. This parameter represents the volume ratio of region of active layer affected by the light absorption of K-AgNPs to the region of active layer.

### 3.3 Optical properties of A-AgNPs and active layer

**Figure 5:**
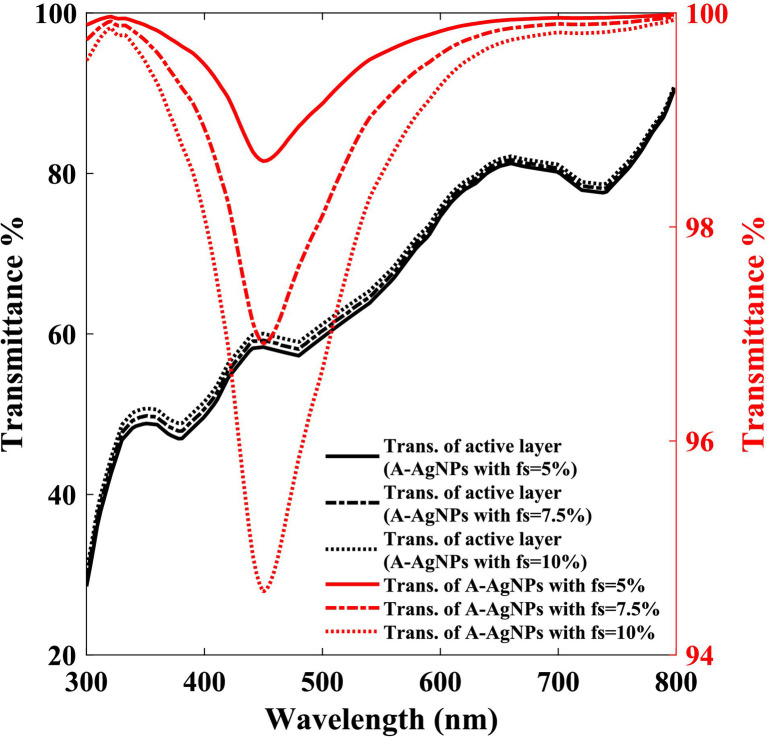
Light transmittance through the A-AgNPs (right scale), and through the bare active layer (left scale) with different filling fraction (f_s_) of A-AgNPs, versus the wavelength.

We investigate the light transmittance within the photovoltaic cell layers to determine the light intensity illuminating the K-AgNPs and to define their light absorption and free carrier generation (Eqs. 9, 10). By applying the Beer–Lambert law to the bare active layer and employing the molar absorptivity method for the A-AgNPs along with the absorption cross-section spectrum (Eq. 7), the light transmittance through these layers across the incident wavelength for different f_s_ values of the A-AgNPs is determined (Figure 5).

Considering a constant volume for a photovoltaic cell, as the f_s_ of the A-AgNPs increased, the volume of the bare active layer decreased slightly. Therefore, with an increase in the f_s_ of the A-AgNPs, the light transmittance through the A-AgNPs is decreased, slightly increasing the transmittance of the bare active layer owing to its volume reduction. Figure 5 exhibits a light absorption in the wavelength of 453 nm which is due to the A-AgNPs resonant absorption. The transmittances of the A-AgNPs for f_s_ values of 5, 7.5, and 10% at incident light wavelength of 453 nm are 99, 97, and 94.6%, respectively. The corresponding light transmittance amplitude for the bare active layer are 56, 57, and 58%, respectively. The multiplication of transmittance of the bare active layer and A-AgNPs is equal to T_r_ which is used during calculating the coefficient of ‘k’ in Eq. 10. Multiplication of T_r_ and incident light intensity is the radiated light to the K-AgNPs.

### 3.4 Photovoltaic cell performance with A-AgNPs and K-AgNPs

**Figure 6:**
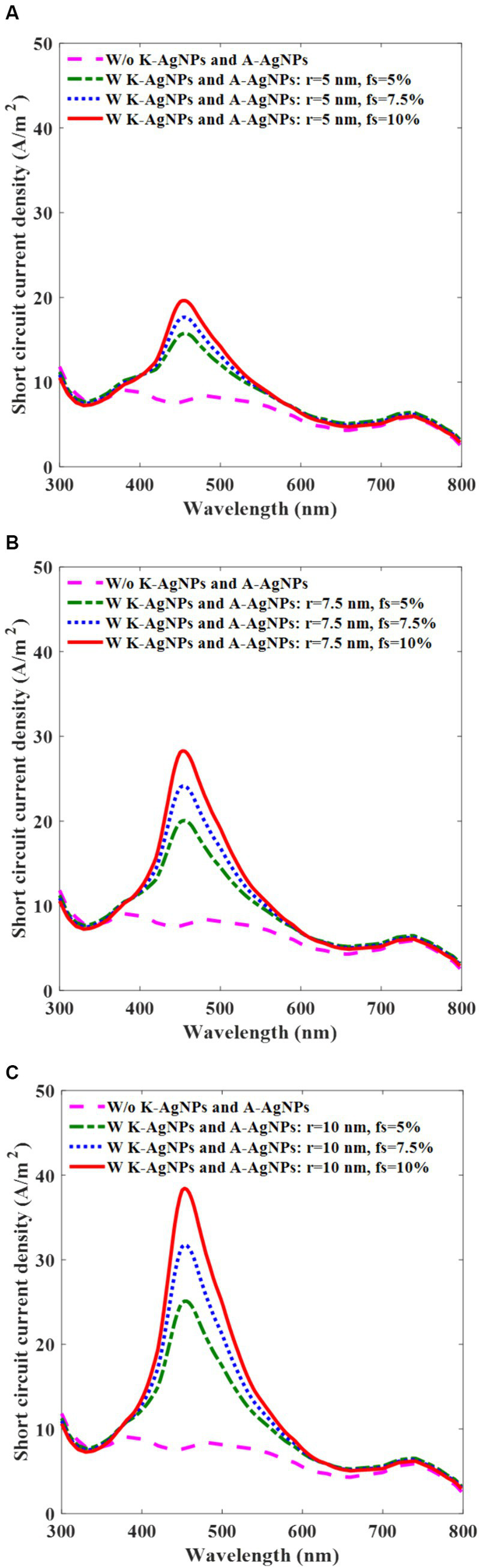
Photovoltaic cell short-circuit current density (J_sc_) with (w) and without (w/o) K-AgNPs and A-AgNPs in the active layer, with intensity of 0.26 mW/mm^2^ for the various filling fractions (f_s_) of A-AgNPs and for different radii of 5 nm **(A)**, 7.5 nm **(B)**, and 10 nm **(C)**. The radius and the interparticle gap of K-AgNPs are, 25 nm and 1.5 nm, respectively.

Since current generation is the main parameter used to specify the PCE of a photovoltaic cell, we investigated the effect of using A-AgNPs and K-AgNPs on the current generation of the device over the incident wavelength. Figure 6 reveals that when a photovoltaic cell is irradiated by a light intensity of 0.26 mW/mm^2^, J_sc_ of the embedded photovoltaic cell peaks at the LSPR wavelength. For the photovoltaic cell incorporated with K-AgNPs, and A-AgNPs having a radius of 5 nm and f_s_ of 5, 7.5, and 10%, the J_sc_ peak values are 15, 17, and 20 A/m^2^, respectively at the wavelength of 453 nm (Figure 6A). For the A-AgNPs with the radii of 7.5 and 10 nm, the corresponding values for J_sc_ are 20, 24, and 29 A/m^2^ and 25, 32, and 39 A/m^2^, respectively (Figures 6B,C). Based on Eq. 10, the J_sc_ is proportional to the concentration of the A-AgNPs in the photovoltaic cell volume and hence, the J_sc_ is significantly enhanced by increasing the radius and f_s_ of the A-AgNPs. Figures 6B,C show that for the bigger radius of the A-AgNPs, with increasing the f_s_, the J_sc_ is higher and peaks to the higher values. This can be attributed to the higher absorption of larger A-AgNPs at higher concentrations. The results show that incorporating A-AgNPs in the active layer and K-AgNPs as a cathode, generates a higher J_sc_ at resonance wavelength compared to the bare active layer (8 A/m^2^).

To achieve high efficiency, we chosse A-AgNPs with a radius of 10 nm and f_s_ of 10% as the preferred configuration. Based on Figure 6, the A-AgNPs with radius of 10 nm and fs of 10% have higher current generation in contrast to the A-AgNPs with the radii of 5 and 7.5 nm, under the same irradiance of 0.26 mW/mm^2^. Selecting a size and f_s_ more than these values damages the active layer function (You et al., 2014). The radius and interparticle gap of the K-AgNPs in all simulations are 25 and 1.5 nm, respectively.

Simulation of the I-V curve provides a precise evaluation of the photovoltaic cell function. Two distinct conditions are explored for the I-V simulation: One involves A-AgNPs with a radius of 7.5 nm and light intensity of 0.33 mW/mm^2^, while the other features A-AgNPs with a radius of 10 nm and a light intensity of 0.26 mW/mm^2^.

**Figure 7:**
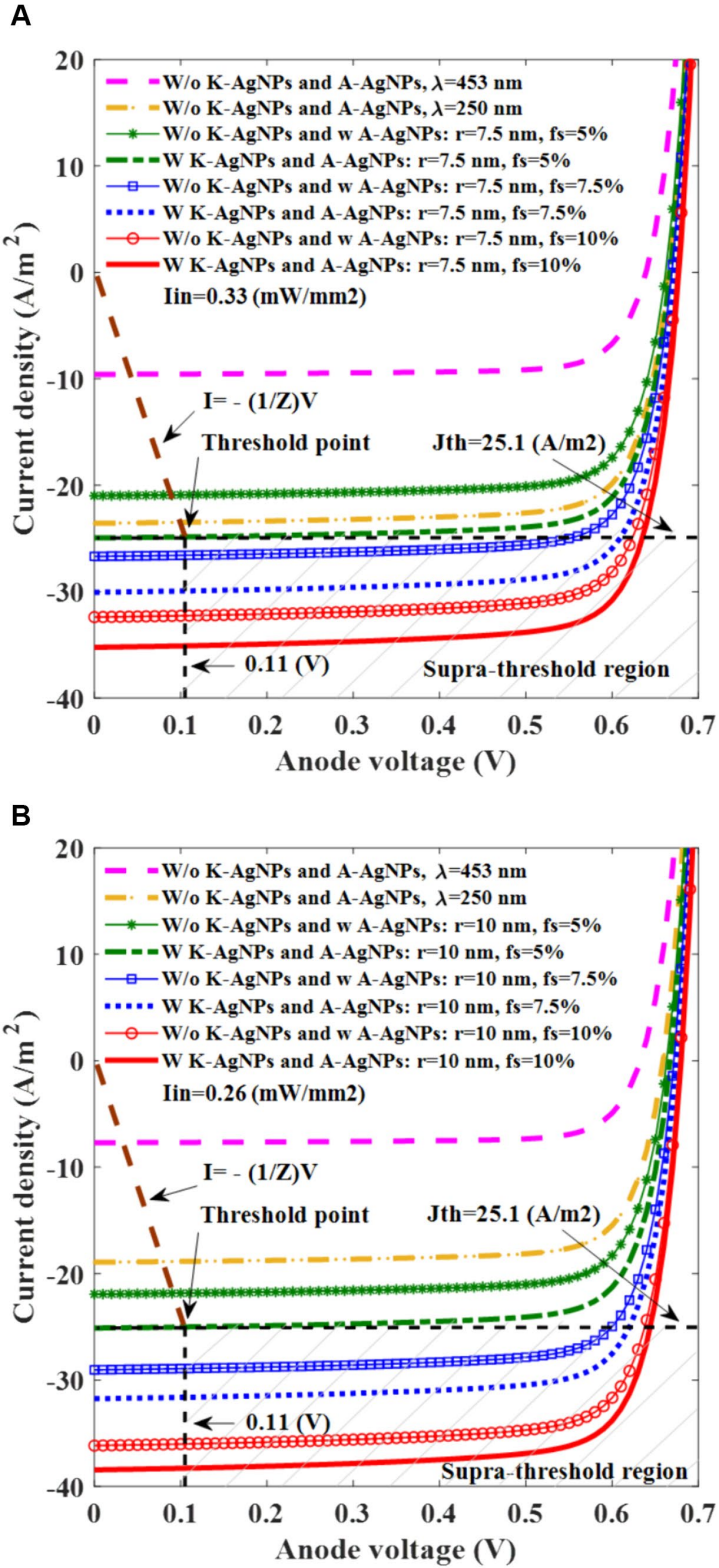
I-V characteristic of photovoltaic cell for different filling fractions (f_s_) for silver nanoparticles in the active layer (A-AgNPs) at 453 nm incident light wavelength with a radius of 7.5 nm and the illumination of 0.33 mW/mm^2^ **(A)**, and A-AgNPs with a radius of 10 nm and the illumination of 0.26 mW/mm^2^ **(B)**. The radius and the interparticle gap of K-AgNPs are 25 nm and 1.5 nm, respectively.

Figure 7A shows that the photovoltaic cell without K-AgNPs and with A-AgNPs in the active layer with a radius of 7.5 nm for f_s_ values of 5, 7.5, and 10% has a J_sc_ of 22, 27, and 33 A/m^2^, respectively, whereas the devices with K-AgNPs and A-AgNPs has a J_sc_ of 25, 30, and 36 A/m^2^. This indicates that the K-AgNPs increased 10% of the J_sc_. Additionally, for the case for f_s_ of 10% with K-AgNPs and A-AgNPs, J_sc_ is 36 A/m^2^, which is 1.5 times higher than that of the bare active layer at a wavelength of 250 nm with a J_sc_ of 24 A/m^2^. A wavelength of 250 nm is the pivotal point for the maximum light absorption and generation rate of bare PCPDTBT:PCBM, providing a suitable control curve for comparison with the NP-embedded photovoltaic cells. Accordingly, the photovoltaic cells without K-AgNPs and with A-AgNPs with a radius of 10 nm for f_s_ values of 5, 7.5, and 10% has the J_sc_ of 23, 29, and 36 A/m^2^ and for the case of K-AgNPs and A-AgNPs, J_sc_ values of 25, 32, and 39 A/m^2^. In this configuration, an increase of 10% in J_sc_ is observed in the case with K-AgNPs, in contrast to the case without them. Here, for the A-AgNPs with f_s_ of 10% and incorporating both K-AgNPs and A-AgNPs, J_sc_ is 39%, which is two times more than the J_sc_ of the bare active layer at a wavelength of 250 nm (19 A/m^2^). Figures 7A,B significantly represent the role of K-AgNPs, light intensity, radius, and f_s_ of A-AgNPs in carrier generation at this interface. Both graphs show that for the bare active layer at a wavelength of 453 nm, J_sc_ is weak and less than 10 A/m^2^.

The I-V characteristics of the electrode-electrolyte interface are obtained to determine the actual current injected through the electrode connected to the photovoltaic cell. The electrode–electrolyte impedance (Z) is calculated based on the threshold current and voltage mentioned in Section 2.4. The I-V curve that represents the electrode-electrolyte interface is plotted with a slope of −1/Z, as shown in the brown dotted curve in Figure 7.

Subsequently, using a photovoltaic cell, we identified the threshold light intensity required to initiate neural activation. As the photovoltaic cell is connected to the electrode in series, the intersection point of the I-V curves of the photovoltaic cell and the electrode-electrolyte interface indicates the current and voltage applied to the neuron. Figure 7 show that the photovoltaic cell with K-AgNPs and A-AgNPs with f_s_ ≥ 5% and radii of 7.5 and 10 nm generate the threshold current density necessary to activate the neuron with light intensities of 0.33 and 0.26 mW/mm^2^, respectively.

**Figure 8:**
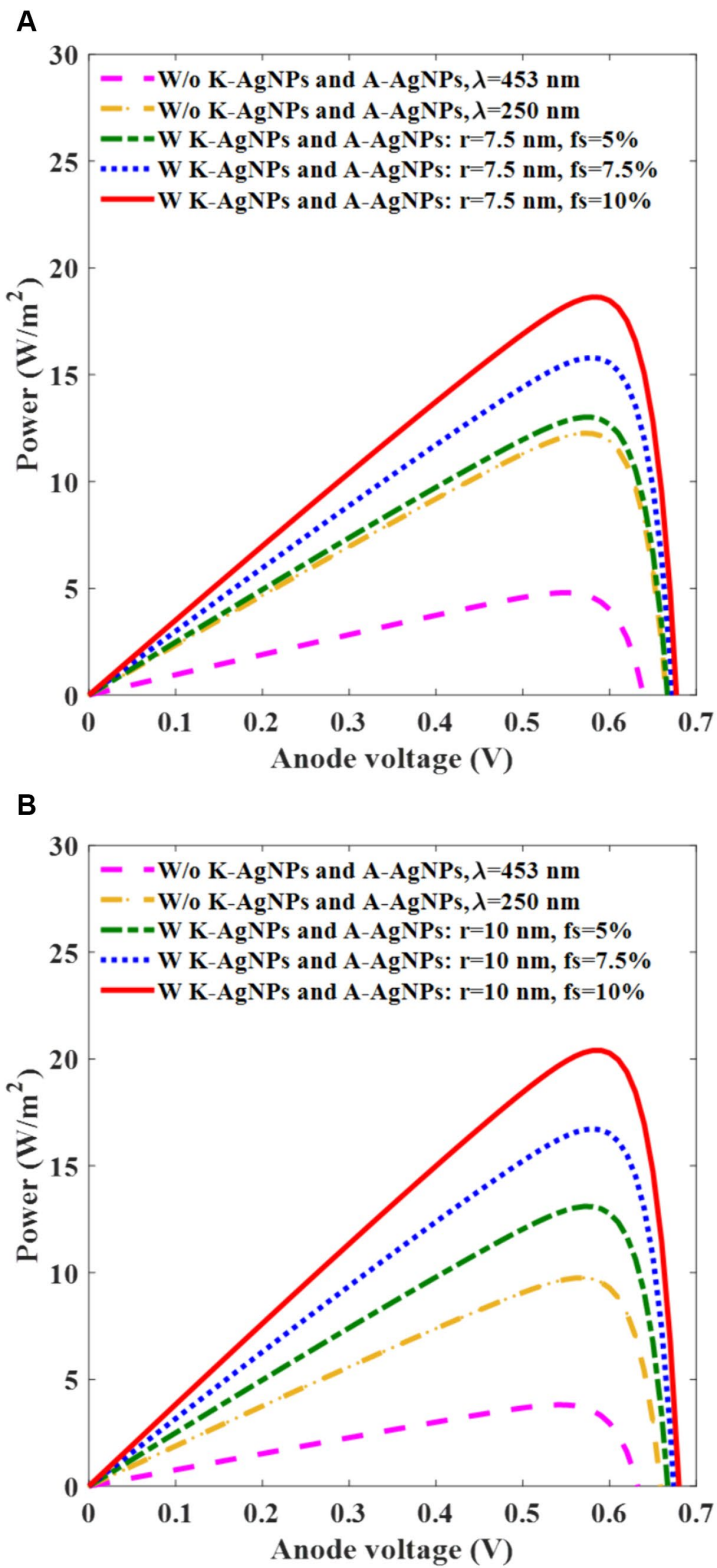
Power-voltage graph of photovoltaic cell with (w) and without (w/o) K-AgNPs and A-AgNPs, for various filling fractions (f_s_) of A-AgNPs in the active layer at 453 nm wavelength for A-AgNPs radius of 7.5 nm and light intensity of 0.33 mW/mm^2^ **(A)**, A-AgNPs with a radius of 10 nm and light intensity of 0.26 mW/mm^2^ **(B)**. The radius and the interparticle gap of K-AgNPs are 25 nm and 1.5 nm, respectively.

The power characteristics of the photovoltaic cell versus the anode voltage are investigated (Figure 8) to determine the performance of the device. This analysis is conducted at 453 nm incident light wavelength and for different f_s_ of A-AgNPs and radii of 7.5 nm and 10 nm under irradiation of 0.33 mW/mm^2^ and 0.26 mW/mm^2^, respectively (Figures 8A,B). In Figure 8A, for the active layer with A-AgNPs and f_s_ of 5, 7.5, and 10%, the power is 13, 16.5, and 18.5 W/m^2^ which surpasses those obtained without K-AgNPs and A-AgNPs at both 453 and 250 nm wavelengths, having power of 5, and 12 W/m^2^, respectively. For A-AgNPs with a radius of 10 nm, the corresponding powers are 13, 17, and 21 W/m^2^, exceeding the values obtained without AgNPs at wavelengths of 453 and 250 nm with powers of 4 and 10 W/m^2^, respectively.

These results highlight the significant role of A-AgNPs and K-AgNPs in comparison with bare photovoltaic cells. The performance parameters of the photovoltaic cell were extracted and are listed in Table 4. The fill factor (FF) is computed as P_max_/I_sc_V_sc_ and the PCE is extracted from P_max_/P_in_, where P_max_ equals I_max_ × V_max_, P_in_ is A × I_in_, with I_in_ representing the incident light intensity, and A indicates the photovoltaic cell area. Analysis of the results in Table 4 reveals a direct relationship between the increase in the PCE of the device and the increase in the f_s_ and radius of the A-AgNPs. Specifically, the PCE ratio of the embedded photovoltaic cell with K-AgNPs and A-AgNPs with radius of 7.5 nm and f_s_ of 10% to the bare photovoltaic cell at a wavelength of 250 nm is 1.5. Similarly, for A-AgNPs with a radius of 10 nm and f_s_ of 10%, the ratio is 2.

**Table 4:** Photovoltaic cell performance parameters for different configurations.

Photovoltaic cell configuration	A-AgNPs’ radius (nm)	I_in_ (mW/mm^2^)	J_sc_ (A/m^2^)	V_oc_ (V)	FF (%)	PCE (%)
Active layer w/o K-AgNPs and A-AgNPs, λ = 453 nm	–	0.33	9.5	0.67	78.5	1.5
	–	0.26	7.5	0.67	75.5	1.4
Active layer w/o K-AgNPs and A-AgNPs, λ = 250 nm	–	0.33	23	0.68	78	3.5
	–	0.26	19	0.68	77.5	3.8
Active layer w K-AgNPs and A-AgNPs: f_s_ = 5%	7.5	0.33	25	0.68	76.5	4
	10	0.26	25	0.68	76.5	5
Active layer w K-AgNPs and A-AgNPs: f_s_ = 7.5%	7.5	0.33	30	0.685	78	4.8
	10	0.26	32	0.685	75.2	6.3
Active layer w K-AgNPs and A-AgNPs: f_s_ = 10%	7.5	0.33	35	0.69	76.5	5.6
	10	0.26	38	0.69	80	8

### 3.5 Neural stimulation by plasmonic photovoltaic cells

**Figure 9:**
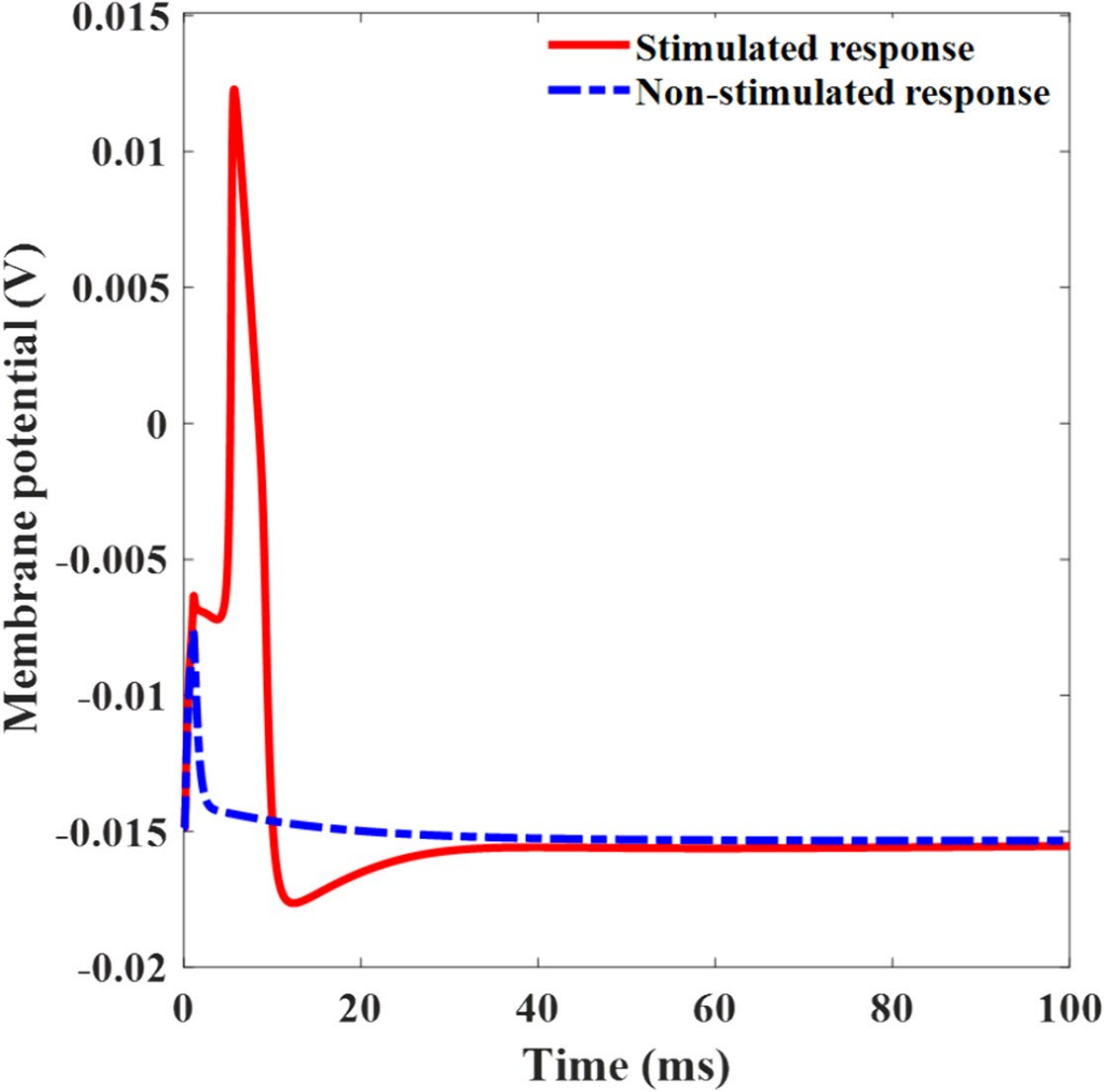
Membrane potential for stimulated neuron response with the input voltage of −0.11 V, and non-stimulated neuron response with the under-threshold input voltage of −0.10 V. The input pulse duration is 1 ms.

In this study, we investigated retinal neural activation using plasmonic solar radiation. Upon illuminating light at the threshold light intensity (0.26 mW/mm^2^) with a duration of 1 ms, the plasmonic photovoltaic cell generates a cathodic potential pulse of −0.11 V, eliciting an action potential. Conversely, when shining light with an intensity lower than 0.26 mW/mm^2^, the voltage applied to the electrode becomes −0.10 V and the neuron remains silent (Figure 9). Notably, this potential resides within the water window, precluding water oxidation, and ensuring that neuronal stimulation at this potential does not result in electrode degradation (Boinagrov et al., 2016). Furthermore, as indicated in Table 4, the maximum cathode voltage is 0.69 V, which is well within the water window range of 1.23 V, ensuring that electrolytic neural stimulation did not occur. To mitigate this possibility, it is crucial to determine the voltage applied to the electrode precisely and constrain it within the water window.

## 4 Discussion

In this study, we explored a structural approach for enhancing the efficiency of a photovoltaic cell as a PV interface in a retinal prosthesis. Our findings illustrate that the incorporation of A-AgNPs into the active layer significantly improves the PCE of the photovoltaic cell compared with its bare counterpart. Moreover, substituting the K-AgNPs with the cathode plate further elevated the PCE compared with the A-AgNP-embedded active layer alone. To ascertain whether the designed structure could effectively stimulate retinal neurons, a SIM4LIFE LIGHT simulator is employed to define the threshold levels of current density and applied voltage required for neuronal activation.

In this study, we modeled the comb-shaped configuration of the active layer to simulate its structure. However, we used this modeling only as a simulation modeling, there are research groups fabricate the comb shape structure to get higher efficiency in practical cases (Aryal et al., 2009). Since we used metal nanoparticle in both active layer (A-AgNPs) and cathode (K-AgNPs), the main limitation is the work function of metal for K-AgNPs to connect to the active layer. The conventional biomedical metals with allowed work function to connect to the PCPDTBT:PCBM active layer are, Ti, Al, and Ag. Among these metals, only A-AgNPs resonance wavelength is in the visible band and other metals resonance are out of visible and IR range. Although, there is a potential for corrosion of Ag metal over the long term, there are numerous studies have investigated the utilization of AgNPs in biomedical applications (Shanmuganathan et al., 2019; Ankush et al., 2022; Naganthran et al., 2022). This suggests that AgNPs not as a bulk metal may offer a viable option for long-term biomedical application.

As the A-AgNPs and K-AgNPs should have the same resonance, the size and interparticle gap of the K-AgNPs are adjusted to achieve the same plasmonic resonance wavelength as the resonance of A-AgNPs in the active layer, as well as the incident light wavelength (453 nm). Simulations incorporated K-AgNPs with a radius of 25 nm and an interparticle gap of 1.5 nm, ensuring their resonance adaptation. This wavelength is useful for patients who have missed their natural visual sensitivity completely (Ferlauto et al., 2018; Airaghi Leccardi et al., 2020). However, positioning the nanoparticles in the specific points in the range of sub-nanometer on the substrate remains a problem. In this regard, there are many researches in which the growth and position of the nanoparticle *in situ* is controllable (Chai et al., 2010).

We simulated photovoltaic cells with- and without- spherical A-AgNPs and K-AgNPs and extracted the J_sc_ and I-V curves of the device. The organic material chosen for the active layer was PCPDTBT:PCBM, which is recognized for its kind carrier properties in bulk heterojunction formation, in contrast to some conventional organic materials such as P3HT:PCBM.

The plasmonic effect of the A-AgNPs in the active layer and the far-field optical properties of the K-AgNPs were thoroughly examined. These findings indicate that for small A-AgNPs, the absorption term dominates, whereas the scattering term is negligible. These insights are crucial for calculating J_sc_. The J_sc_ and I-V curves were analyzed for varying radii and f_s_ of the A-AgNPs to identify a suitable configuration of the device. The I-V graph data reveal that the inclusion of K-AgNPs enhances the current density of the device by 10% compared to its absence. The results demonstrated a direct proportionality between the Jsc of the photovoltaic cells and the size and f_s_ of the A-AgNPs. Analysis of the plasmonic effect and Jsc reveal that the LSPR of the A-AgNPs occurs at a wavelength of 453 nm, and heightened carrier generation is observed for A-AgNPs with radii of 7.5 nm and 10 nm. Our preferred configuration involves A-AgNPs with a radius of 10 nm and f_s_ of 10%, primarily owing to its lower light intensity (0.26 mW/mm^2^) which leads to 39 A/m^2^, compared to that of a radius of 7.5 nm (0.33 mW/mm^2^). In comparison with the presented works that have used quantum dots for improving the photovoltaic function, incorporating quantum dots have the advantages of high absorption coefficient for thin active layer and size tunable bandgap through the quantum confinement effect which can result in NIR absorption wavelength (Karatum et al., 2022). Although, using high input intensity of 1 mW/mm^2^ leads to the current density of 550 μA/cm^2^ and 5.5 mA/W responsivity, which is lower than conventional responsivity for organic photovoltaic cells (Airaghi Leccardi et al., 2020; Chenais et al., 2021).

The inclusion of larger nanoparticles results in exciton quenching and carrier trapping, whereas an f_s_ exceeding 10% can be detrimental to the performance of the active layer. These configurations are fine-tuned to satisfy the threshold conditions required for neuronal activation. Power-voltage graphs facilitate the computation of the performance parameters of the photovoltaic cell. According to the data presented in Table 4, these parameters confirm that our preferred configuration results in a favorable FF and PCE.

## 5 Conclusion

A structural technique was employed to design a high-efficiency photovoltaic-based retinal prosthesis. In addition to embedding spherical A-AgNPs into the active layer, K-AgNPs were introduced as cathode electrode, serving dual roles as both the cathode and plasmonic nanoparticles. The resonance wavelengths of both nanoparticle types were aligned through meticulous device configuration adjustments. Upon irradiation of the device with incident light tuned to an LSPR wavelength of 453 nm, the total absorption of the photovoltaic cell was significantly enhanced. This structural technique results in higher carrier generation and efficiency.

Our preferred option as the ultimate configuration of the photovoltaic cell involves K-AgNPs with a radius of 25 nm, an interparticle gap of 1.5 nm, A-AgNPs with a radius of 10 nm, and a f_s_ of 10%, illuminated with a light power of 0.26 mW/mm^2^, tuned on 453 nm. This specific structural arrangement led to a PCE ratio of 2 compared to the bare photovoltaic cell at the maximum light absorption wavelength of PCPDTBT:PCBM at 250 nm. The incorporation of K-AgNPs played a crucial role, contributing to a 10% increase in the carrier generation. This implies that the photovoltaic cell dimensions and incident light intensity can be effectively minimized using this ratio. Simulation results unequivocally establish the efficacy of the proposed structure as an efficient technique for enhancing photovoltaic-based retinal prostheses.

## Data availability statement

The raw data supporting the conclusions of this article will be made available by the authors, without undue reservation.

## Author contributions

AR: Conceptualization, Data curation, Formal analysis, Investigation, Methodology, Software, Validation, Visualization, Writing – original draft, Writing – review & editing. KE: Conceptualization, Funding acquisition, Investigation, Methodology, Supervision, Writing – original draft, Writing – review & editing.
